# Wet Markets and Food Safety: TripAdvisor for Improved Global Digital Surveillance

**DOI:** 10.2196/11477

**Published:** 2019-04-01

**Authors:** Nicole E Kogan, Isabelle Bolon, Nicolas Ray, Gabriel Alcoba, Jose L Fernandez-Marquez, Martin M Müller, Sharada P Mohanty, Rafael Ruiz de Castañeda

**Affiliations:** 1 Massachusetts Institute of Technology Cambridge, MA United States; 2 Institute of Global Health Faculty of Medicine University of Geneva Geneva Switzerland; 3 Institute for Environmental Sciences University of Geneva Geneva Switzerland; 4 Division of Tropical and Humanitarian Medicine University Hospitals of Geneva Geneva Switzerland; 5 Citizen Cyberlab Centre Universitaire d’Informatique University of Geneva Carouge Switzerland; 6 Digital Epidemiology Lab École Polytechnique Fédérale de Lausanne Lausanne Switzerland

**Keywords:** epidemiology, maps, foodborne diseases, social networking, travel, agriculture

## Abstract

**Background:**

Wet markets are markets selling fresh meat and produce. Wet markets are critical for food security and sustainable development in their respective regions. Due to their cultural significance, they attract numerous visitors and consequently generate tourist-geared information on the Web (ie, on social networks such as *TripAdvisor*). These data can be used to create a novel, international wet market inventory to support epidemiological surveillance and control in such settings, which are often associated with negative health outcomes.

**Objective:**

Using social network data, we aimed to assess the level of wet markets’ touristic importance on the Web, produce the first distribution map of wet markets of touristic interest, and identify common diseases facing visitors in these settings.

**Methods:**

A *Google* search was performed on 31 food market–related keywords, with the first 150 results for each keyword evaluated based on their relevance to tourism. Of all these queries, *wet market* had the highest number of tourism-related *Google Search* results; among these, *TripAdvisor* was the most frequently-occurring travel information aggregator, prompting its selection as the data source for this study. A Web scraping tool (*ParseHub*) was used to extract wet market names, locations, and reviews from *TripAdvisor*. The latter were searched for disease-related content, which enabled assignment of *GeoSentinel* diagnosis codes to each. This syndromic categorization was overlaid onto a mapping of wet market locations. Regional prevalence of the most commonly occurring symptom group - food poisoning - was then determined (ie, by dividing the number of wet markets per continent with more than or equal to 1 review containing this syndrome by the total number of wet markets on that continent with syndromic information).

**Results:**

Of the 1090 hits on *TripAdvisor* for *wet market*, 36.06% (393/1090) conformed to the query’s definition; wet markets were heterogeneously distributed: Asia concentrated 62.6% (246/393) of them, Europe 19.3% (76/393), North America 7.9% (31/393), Oceania 5.1% (20/393), Africa 3.1% (12/393), and South America 2.0% (8/393). Syndromic information was available for 14.5% (57/393) of wet markets. The most frequently occurring syndrome among visitors to these wet markets was food poisoning, accounting for 54% (51/95) of diagnoses. Cases of this syndrome were identified in 56% (22/39) of wet markets with syndromic information in Asia, 71% (5/7) in Europe, and 71% (5/7) in North America. All wet markets in South America and Oceania reported food poisoning cases, but the number of reviews with syndromic information was very limited in these regions (n=2).

**Conclusions:**

The map produced illustrates the potential role of touristically relevant social network data to support global epidemiological surveillance. This includes the possibility to approximate the global distribution of wet markets and to identify diseases (ie, food poisoning) that are most prevalent in such settings.

## Introduction

### Background

Traditional food markets (ie, wet markets) play important roles in food security and local development [1]; however, they also have negative health implications. In 2003, severe acute respiratory syndrome spread globally from a Chinese wet market, causing hundreds of deaths [2] and major economic losses [3]. Avian influenza has also been repeatedly associated with wet markets [1,4]. Foodborne *Campylobacter*, *Salmonella*, *Giardia*, and *Escherichia* are most common in these settings, leading to 18 million disability-adjusted life years annually [5]. These are particularly important in low- and middle-income countries (LMICs), but their true impact is unknown given that many episodes go unreported [6].

Due to wet markets’ cultural importance, there exists extensive tourist-geared information on the Web, often on websites such as *Yelp* and *TripAdvisor*, which serve as forums to share experiences. Although the use of these data remains unexploited for wet markets, it has yielded compelling results for restaurants. For example, *iwaspoisoned.com* [7] serves as a platform where individuals can report symptoms of food poisoning alongside the offending eatery. Through citizen participation, the website has identified several foodborne disease outbreaks before traditional epidemiological methods [8].

### Objectives

This approach shaped the objectives of this study, which were to show the link between wet markets and tourism as well as to exploit tourist-generated social network data to create the first map of the distribution of wet markets of touristic interest and their associated adverse health events. Rather than function as an epidemiological analysis in which foodborne disease incidence related to wet market visits is calculated, our approach aims to showcase the potential of Web-based social networks to pick up potentially overlooked instances of disease.

## Methods

### Establishing the Data Source

A *Google* search was performed on different food market types (see Multimedia Appendix 1). For each keyword, the first 150 results were scraped and characterized based on their relevance to tourism (ie, presence of tourist-geared content and promotion of an area to potential visitors). Of all food market types, *wet market* was linked to the highest proportion of tourism-related websites—59.3% (89/150) of *Google Search* results. This connection motivated its use in this study. Any social networking websites were flagged, and their touristic importance was assessed. Of these, *TripAdvisor* appeared most frequently and had the most comprehensive wet market–related information, prompting its selection as this study’s data source.

### Web Scraping

The term *wet market* was inputted into *TripAdvisor* (July 2017). Wet market names and locations were harvested using *ParseHub* [9]. Irrelevant results (eg, waterparks with *wet* in their names) were removed manually. A *Python 2.7* script integrating geocoding library *GeoPy* [10] was developed to convert wet market locations into geographic coordinates for mapping.

### Text Mining

For each wet market, the *TripAdvisor* *Reviews* section was parsed for mentions of keywords most often associated with foodborne disease, a list corroborated by the National Institutes of Health National Institute of Diabetes and Digestive and Kidney Diseases Web page on foodborne illness [11]: *diarrhea*, *vomit/vomiting/vomited*, *food* *poisoning*, *stomach ache*, *headache*, *nausea/nauseous*, *upset stomach*, *sick*, *ill*, and *dizzy*. Comments containing at least one of these were manually extracted. When possible, comments were assigned a *GeoSentinel* diagnosis code [12] through medical expert analysis using syndrome keywords and indicators of symptom duration. Wet market and syndromic distribution were analyzed using descriptive statistics.

The study represents a passive analysis of information on *TripAdvisor*. The investigators did not participate in nor were involved in *TripAdvisor* communications (ie, there was no posting), so the analysis should not be considered active internet-based research requiring human subject consent.

## Results

### Global Wet Market Distribution

**Figure 1:**
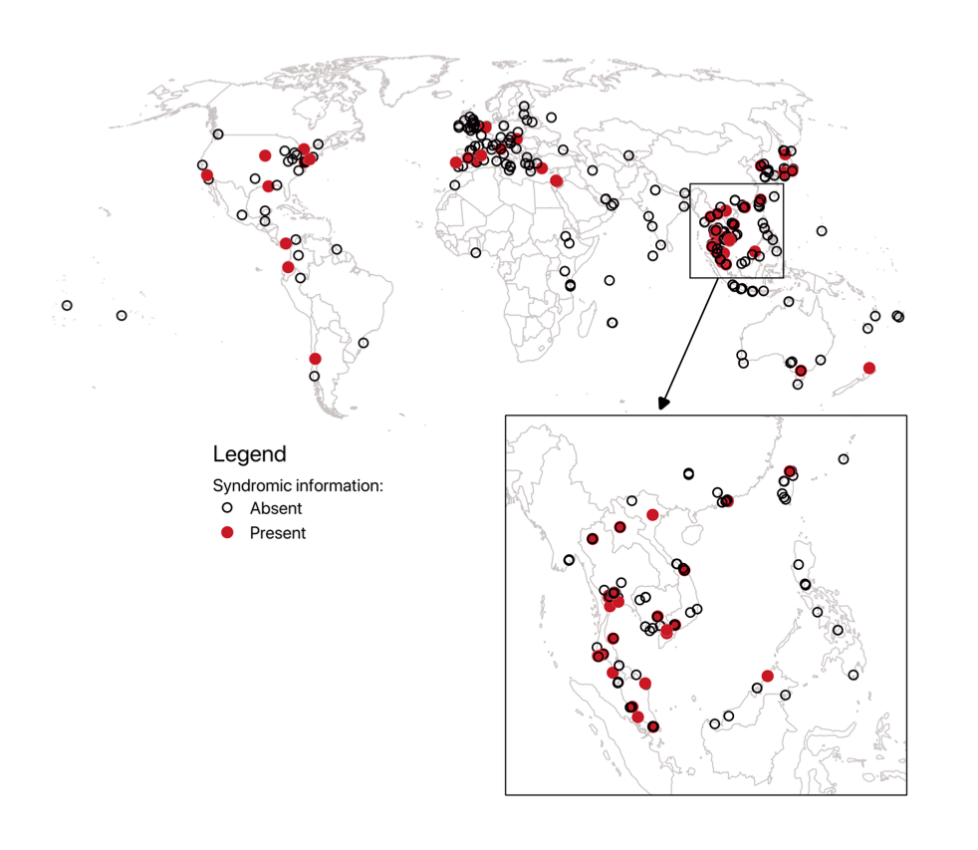
Locations of TripAdvisor-sourced wet markets, with zoomed inset in Southeast Asia. Red circles represent wet markets where visitors reported adverse health events, whereas empty circles denote locations where such reports were lacking. The background country border is sourced from Natural Earth vector data projected in the World Robinson coordinate reference system using Quantum Geographic Information System (QGIS) 2.18.2.

*Wet market* yielded 1090 attractions on *TripAdvisor*, 36.06% (393/1090) of which aligned with the term’s definition. Mapping revealed Asia as the region with the greatest wet market density, accounting for 62.6% (246/393) of wet markets reviewed (Figure 1). The second-most wet market–dense region was Europe with 19.3% (76/393) of wet markets reviewed, followed by North America with 7.9% (31/393), Oceania with 5.1% (20/393), Africa with 3.1% (12/393), and South America with 2.0% (8/393) *.*

### Assessment of Syndromic Information

**Figure 2:**
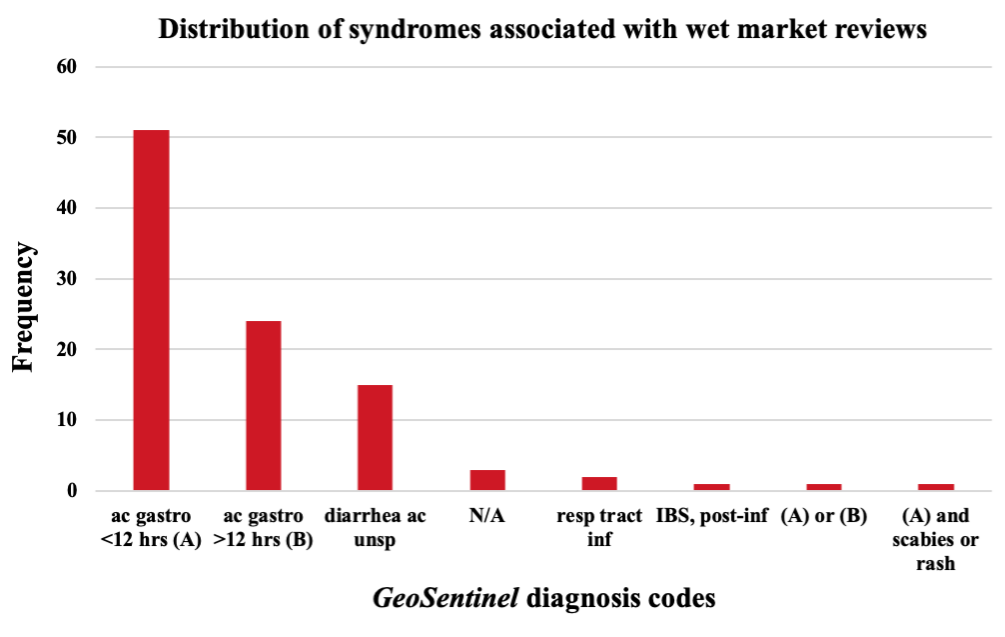
Frequency distribution of 98 TripAdvisor reviewer diagnoses following GeoSentinel encoding. ac=acute; gastro=gastroenteritis; IBS=irritable bowel syndrome; N/A=symptoms present but illness unascertainable; resp tract inf=respiratory tract infection; unsp=unspecified.

All 393 wet markets were reviewed on *TripAdvisor*. Moreover, 14.5% (57/393) of these contained at least one review with syndromic information and were broken down regionally as 68% (39/57) Asian, 12% (7/57) European, 12% North American (7/57), 3% South American (2/57), and 3% Oceanic (2/57). In total, these yielded 98 reviews with syndromic information, of which Asian markets accounted for 74% (73/98). *Acute gastroenteritis <12 hrs, food poisoning* was the most common diagnosis in wet markets globally 54% (51/95) syndrome references, after discarding 3 reviews corresponding to unascertainable illness; Figure 2). Asia, where the review volume was particularly noticeable, had 56% (22/39) of wet markets with this specific syndrome. The syndromes *Acute gastroenteritis > 12 hrs*, *Acute gastroenteritis <12 hrs*, and *Diarrhea, acute unspecified* comprised 95% (90/95) non-*N/A* diagnoses from the 57 wet markets.

## Discussion

### Principal Findings

This study shows the touristic importance of wet markets and provides, to our knowledge, the first global map of wet markets’ locations and related syndromic information communicated on *TripAdvisor* by their visitors. Our map reveals that wet markets are heterogeneously distributed across continents. Asia is a hotspot, both in the number of markets and the absolute number of adverse associated health events. When controlling by the number of continental wet markets with syndromic information, South America and Oceania exhibited the highest proportion of *GeoSentinel* *acute gastroenteritis <12 hrs, food poisoning* diagnoses. However, both these regions were wet market–sparse (n=2 wet markets with syndromic information in each) compared with a continent like Asia.

Use of region-specific terminology for designating markets could have yielded denser mapping and should be used in future work. Furthermore, the choice to look only at *TripAdvisor* reviews in English may have impacted the wet market distribution shown in the map. An example is the density of wet markets, which seems to be in preferred destinations of English-speaking tourists; another example is that the concentration of wet markets in Africa is largely in English-speaking countries. This could prompt search on the platform using other languages to enhance the results.

Little is known about the wet market health threats to locals and travelers partly because of underreporting of adverse health events experienced by visitors [13]. Although social networking platforms do not paint a complete picture of wet markets (eg, total number of regional wet markets and how many yearly visitors), they can provide opportunities to instantaneously report and detect these elusive cases—primarily traveler-related ones—and improve epidemiological monitoring within these settings.

### Limitations and Strengths

Reviews from *Yelp*, a *TripAdvisor*-like platform connecting citizens with local businesses, have been leveraged by New York public health authorities to detect restaurant-related foodborne disease events [14]; interestingly, fewer than 2% of individuals with an alleged illness explicitly mention reporting their case to a medical professional [14]. *Twitter* has been implemented in St. Louis, Missouri, to detect food poisoning cases [15], but its potential is restricted by the short post length. The website *iwaspoisoned.com* is equally notable in its effort to crowdsource information, though it is limited in scope outside the United States. Wet markets are more challenging establishments because of their typical location in LMICs and their operation under often-limited regulations. This necessitates tapping into other Web-based communities—social platforms such as *TripAdvisor—* to glean information on visitor health.

Traveler’s diarrhea is a common disorder affecting tourists visiting developing countries [16] and is generally associated with consumption of foods prepared under unhygienic conditions (common in wet markets). Our analysis shows that acute gastroenteritis (food poisoning) and diarrhea were the most frequent illnesses among wet market visitors. However, this must be carefully considered from medical and epidemiological perspectives as the result is based on rarely corroborated, Web-based descriptions. We also cannot exclude that reported symptoms could have been caused by an event before or after (but unrelated to) a wet market visit.

### Conclusions

As use of *TripAdvisor* in isolation has accounted for only a fraction of wet markets of touristic importance and their associated health risks, larger datasets are needed to confirm the results presented here and to explore others. Even so, the pipeline we present is significant for travel medicine and epidemiology. It could ultimately contribute to predictive models for improved epidemic forecasting and to the development of diagnostic tools based on syndromic surveillance and artificial intelligence. *TripAdvisor* could crosstalk with other social networks (eg, *Yelp*) for maximal information coverage and partner with other initiatives for a more structured collection of wet market–related health information in near-real time. In this way, we can gain an improved understanding of global wet markets and their associated health risks while also ensuring their safer promotion.

## Appendix

Multimedia Appendix 1 List of market types whose relevance to tourism was assessed in an initial Google Search screen.

## Abbreviations

LMIC: low- and middle-income country
