# Supplementary material for: Wet Markets and Food Safety: TripAdvisor for Improved Global Digital Surveillance
Source: JMIR Public Health Surveill. 2019 Apr 1;5(2):e11477. doi: 10.2196/11477 (PMC6462893; doi:10.2196/11477)
Supplement: Multimedia Appendix 1 [file publichealth_v5i2e11477_app1.docx]

Multimedia Appendix

This is a Multimedia Appendix to a full manuscript published in the J Med Internet Res. For full copyright and citation information see http://dx.doi.org/10.2196/jmir.xxxx

Table 1. List of market types, screened for touristic relevance through *Google Search*

| Food market | Farmers market | Green market | Bushmeat market | Wet market | Livestock market | Poultry market | Local market | Local food market |
| --- | --- | --- | --- | --- | --- | --- | --- | --- |
| Local farmers market | Produce market | Organic market | Fresh produce market | Outdoor market | Fresh market | Green food market | Open food market | Open-air market |
| Covered (indoor) market | Street market | Floating market | Night market | Fish market | Wholesale produce market | Live animal market | Seafood market | Native food market |
| Indigenous food market | Wild food market | Country market | Traditional food market |  |  |  |  |  |
